# Heart disease complicating pregnancy as a leading cause of maternal deaths in LMIC settings: the Sri Lankan experience

**DOI:** 10.1016/j.lansea.2023.100223

**Published:** 2023-06-06

**Authors:** Ayesh Hettiarachchi, Kapila Jayaratne, Chithramalee De Silva, Hemantha Senanayake, Niroshan Lokunarangoda, Suneth Agampodi

**Affiliations:** a Department of Community Medicine, Faculty of Medicine and Allied Sciences, Rajarata University of Sri Lanka, Saliyapura, 5008, Sri Lanka; b Family Health Bureau, Ministry of Health, No: 231, De Saram Place, Colombo 10, Sri Lanka; c Faculty of Medicine, University of Colombo, 25, Kynsey Rd, Colombo, 00800, Sri Lanka; d Department of Medicine & Mental Health, Faculty of Medicine, University of Moratuwa, Bandaranayake Mawatha, Moratuwa, 10400, Sri Lanka; e International Vaccine Institute, Seoul, South Korea

**Keywords:** Heart Disease, Maternal deaths, Pregnancy, Sri Lanka, LMIC, Ending preventable maternal deaths, SDG

## Abstract

**Background:**

Heart disease (HD) is one of the leading indirect causes of maternal deaths worldwide, both in high- and low- and middle-income countries (LMICs). This study aims to describe maternal deaths due to cardiovascular disease complicating pregnancy in Sri Lanka.

**Methods:**

The national Maternal Death Surveillance Response (MDSR) system in Sri Lanka investigates all female deaths during pregnancy and 12 months after delivery. These maternal death investigation records were perused in this analysis. Maternal deaths from 2006 to 2018 with HD complicating pregnancy as the immediate or underlying cause of death were re-coded using the ICD-11 classification.

**Findings:**

Of the 2855 pregnancy-related deaths reported to the MDSR from 2006 to 2018, 1646 (57.7%) were confirmed as maternal deaths. Of those, 284 (17.25%) were attributed to HD complicating pregnancy. The cause-specific maternal mortality ratio due to heart disease from 2006 to 2018 was 7.24 per 100,000 live births. Rheumatic heart disease was the leading cause of HD (60, 21.1%), while cardiomyopathies (59, 20.7%) and congenital anomalies (34, 12.0%) accounted for a sizeable share. Medically contraindicated pregnancies accounted for 54 (19%) deaths. Application of the 3-delay model identified 186 (65.5%) cases with possible delays. Out of all deaths, 158 (55.6%) cases were categorized as preventable.

**Interpretation:**

Preventing maternal mortality from HD in LMICs requires a lifecycle approach with situation-specific interventions and highly specialized care. Community awareness, capacity building related to management, and specific infrastructure development will be key strategies.

**Funding:**

None.

**Research in context**

**Evidence before this study**

Despite global commitments to reduce maternal deaths, the target of reducing the global maternal mortality ratio (MMR) to less than 70 per 100,000 live births (LBs) by 2030 is still far from reach. Sri Lanka has shown an impressive reduction in MMR over 50 years from 1694 per 100,000 LBs in 1947 to 63 per 100,000 LBs in 1997, then to an MMR of less than 40 within the next ten years. But over the past decade, MMR has remained stagnant. The increasing number of maternal deaths due to indirect causes demands a cause-specific approach to prevent maternal deaths. Heart Disease (HD) is reported as the second leading cause of maternal death in Sri Lanka.

**Added value of this study**

Data presented in this study clearly shows that HD is a major underlying cause of maternal deaths in Sri Lanka, accounting for almost one-fifth of confirmed maternal deaths over 13 years starting from 2006. This analysis highlights the delays and health system deficits leading to HD related maternal deaths, which need to be addressed at the policy level. The national-level, complete, expert-validated, and curated dataset was the major strength of this study. Observations from this study could be used in the global maternal health agenda to reshape the strategies achieving ending preventable maternal mortality.

**Implications of all the available evidence**

The available data and evidence generated from this study clearly show the need for strategic changes in maternal care in Sri Lanka. The prevention of HD as a leading cause of maternal deaths requires a lifecycle approach, and dedicated program components need to be added to the national pregnancy care program.

## Introduction

The call for envisioning ‘ending preventable maternal mortality’ (EPMM)^1^ and proposing manifestos for maternal health post-2015^2^ by the global maternal health community were positively responded to by the WHO,^3^ UNICEF, and other UN agencies, many governments, and non-governmental organizations. Reducing the global maternal mortality ratio (MMR) to less than 70 per 100,000 live births (LBs) by 2030 was targeted as a sustainable development goal (SDG), specifically target 3.1.^4^ However, despite global commitments to reduce maternal deaths, the target is still far from reach, with 808 women dying every day due to complications of pregnancy and childbirth.^5^ While 94% of global maternal deaths occur in low- and middle-income countries (LMICs), sub-Saharan Africa and South Asia accounted for approximately 86% of estimated global maternal deaths in 2017. According to the World Health Organization (WHO) definitions and categorization, Maternal (obstetric) deaths are categorized as direct and indirect. Direct obstetric deaths are those resulting from obstetric complications of the pregnancy state (pregnancy, labor, and the puerperium), from interventions, omissions, incorrect treatment, or from a chain of events resulting from any of the above. Indirect obstetric deaths are those resulting from previous existing disease or disease that developed during pregnancy and which was not due to direct obstetric causes but which was aggravated by physiologic effects of pregnancy.

Direct obstetric causes still contribute to 73% of global maternal deaths, with hemorrhage at 27.1%, sepsis at 10.7%, abortion at 7.9%, and embolism at 3.2% as the leading underlying causes.^6^ Nevertheless, indirect causes of maternal deaths are increasingly contributing to maternal deaths, especially with the obstetric transition, a phenomenon in which high maternal mortality is declined from a predominance of direct obstetric causes of maternal mortality to an increasing proportion of indirect causes.^7^ These indirect causes have yet to be prioritized by the global maternal health community to reduce preventable maternal deaths further.^8^ Many countries that have reduced MMR to a reasonable level experience stagnant MMR and struggle to reduce MMR further due to the emergence of indirect causes.^9^ Heart disease (HD) combined with hypertensive disorders complicates 1–4% of pregnancies,^10^ yet it has been the leading cause of maternal deaths in the UK^11^ and many other high-income countries. While hypertensive disorders in pregnancy are the second leading cause of maternal deaths globally, HD complicating pregnancy is emerging as a leading cause of maternal death in several LMICs that have already achieved SDG target 3.1.

All maternal organs and systems in the body adapt to pregnancy. Adaptations occur at different times and stages of pregnancy to accommodate increasing demands. This dynamic process is associated with significant physiological changes in the body to permit adequate uteroplacental circulation for fetal growth and development.^12^ Physiological changes occur secondary to the effects of progesterone and estrogen, produced predominantly by the ovaries in the first 12 weeks of pregnancy and thereafter by the placenta. Each organ system is affected differently by the demands of the growing fetus. Cardiovascular and hematological changes occur early in pregnancy, leading to hyperdynamic circulation. Significant cardiovascular changes occur within the first 8 weeks of pregnancy. The risk of heart failure increases early, with a steady increase up to 24 weeks and a plateau until 30 weeks.^13^ A second peak occurs around delivery. With profound hemodynamic, vascular, hematological, and metabolic changes, pregnancy carries an increased risk of cardiovascular events. However, the diagnosis of HD complicating pregnancy is limited in LMICs^14^ due to a lack of diagnostic facilities, resulting in gross underestimation.

**Fig. 1:**
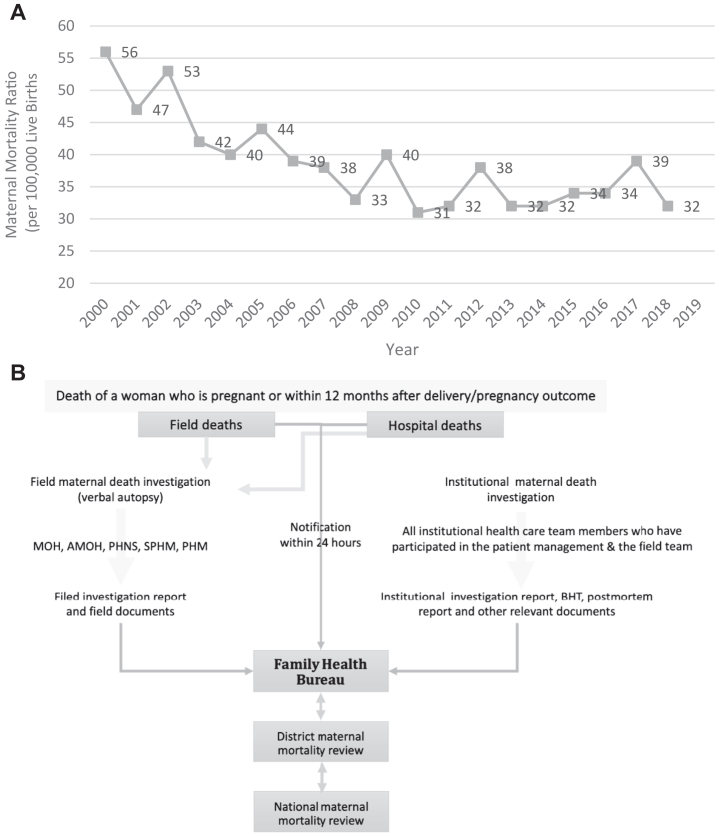
Surveillance-based maternal mortality ratio (MMR) in Sri Lanka (A) and the maternal mortality surveillance system (B).

Sri Lanka has shown an impressive reduction in MMR over 50 years from 1694 per 100,000 LBs in 1947 to 63 per 100,000 LBs in 1997, and then to an MMR of less than 40 within the next ten years. Over the past decade, MMR has remained stagnant, with values of more than 30 per 100,000 live births (Fig. 1A). Being in stage IV of obstetric transition phenomenally, the country struggles to reduce the MMR further. The increasing number of maternal deaths due to indirect causes demands a cause-specific approach to preventing maternal deaths.

In addition, a focus on subnational disparities of MMRs in some districts and an understanding of increasingly indirect causes contributing to maternal mortality are required to develop national policies to reduce MMR. HD is the second leading cause of maternal deaths in Sri Lanka.^15^ National Maternal Mortality Review in 2016^15^ reported 24 deaths due to HD, respectively, contributing to one-fifth of all maternal deaths. The national target of reducing MMR to 10 per 100,000 LBs in 2030,^16^ appears challenging for Sri Lanka.

This paper aims to analyze maternal deaths due to HD complicating pregnancy in Sri Lanka to understand causes and health system deficits, the goal being to provide a case study for the global maternal health community to reshape strategized approaches in achieving EPMM.

## Methods

Data for the study were obtained from the Sri Lanka Maternal Death Surveillance Response (MDSR). All maternal deaths reported to the MDSR system from 2006 to 2018 were included in the analysis.

### Maternal death surveillance system in Sri Lanka

Since its introduction in 1981, the MDSR of Sri Lanka has functioned as one of the most comprehensive systems built into curative and preventive health systems.^17^ Since the issuance of mandatory notification of maternal deaths in 1985, maternal deaths have been reported directly to the Family Health Bureau (FHB). A structured MDSR mechanism is in operation, covering the entire country with data from the community and facility levels.

Sri Lanka uses the definition of the International Classification of Diseases (ICD) application to deaths during pregnancy, childbirth, and the puerperium (ICD-MM) for maternal death as ‘the death of a woman while pregnant or within 42 days of delivery or termination of pregnancy, from any cause related to, or aggravated by the pregnancy or its management, but excluding deaths from incidental or accidental causes’.^18^

When a probable maternal death is known, field and hospital health staff are notified, conduct post-mortem assessments, review the index death at the field and hospital levels, and send an investigation report to the FHB. Any death of a woman while pregnant or up to one year postpartum should be reported to the MDSR system within 24 h of occurrence (Fig. 1B). Both field and hospital investigations are performed using structured formats. Once the investigation process is completed, all reports are sent to the FHB. All maternal deaths are subsequently reviewed at the field, institutional, district, and national levels. At the FHB, the Maternal and Child Morbidity and Mortality Surveillance Unit maintains a database, and comprehensive case scenarios are developed with the available information. These cases are then reviewed by an expert panel of different specialties related to maternal care service provision. A national team of experts from related specialties visits every district in the following year to conduct National Maternal Mortality Reviews (NMMR) at the district level with the participation of all concerned stakeholders. Each maternal death is discussed and reviewed based on 3 delays (deficiencies in seeking health care, accessibility, and treatment). Lessons learned are translated into practice, programs, and policies at the district and national levels.

### Method of data extraction

Hard copies of all maternal death investigation report, bed head tickets, clinical records, pregnancy records, family planning, other field records, and post-mortem reports are available at the FHB for each maternal death. A database with a comprehensive set of variables is maintained. For the present analysis, we used the raw dataset available at the FHB. Any reported maternal death categorized as heart disease as the immediate, intermediate, or underlying cause of death was included in the first round of screening. A careful selection of entries was made at the second screening step to have all cardiovascular diseases documented as the cause of death (COD) during each year's national maternal mortality review. Cerebrovascular conditions were not included in this analysis.

Irrespective of being labeled a maternal death or the death of a woman of reproductive age, all selected entries were recorded using the ICD-11 criteria. First, the deaths were categorized using the ICD-11 by going through the available individual maternal death records. Since the Sri Lankan MDSR is still not using the ICD-11 classification, each entry had to be made separately. Then, once the ICD-11 classification was completed, all maternal deaths were separately recorded using the ICD-11 maternal chapter classification for the cause of death. This also had to be done for each entry as the classification according to the ICD-11 is not routinely done during the national maternal mortality review. Analysis was based on maternal deaths due to heart disease complicating pregnancies, which were filtered out based on the ICD classification.

To compute maternal mortality ratios, the denominator was LBs registered for each district in the year obtained from the Department of Census and Statistics website. Calculations were performed for every 100,000 LBs. Data were presented as proportions and percentages. Missing data were presented in the results as appropriate.

Permission to utilize the national maternal mortality database was obtained from the Director-General of Health Services through the Director-FHB. Anonymized data in electronic format were received for analysis following approval. Ethical clearance was granted by the Ethics Review Committee, Rajarata University of Sri Lanka, for using secondary data from MDSR (ERC/2019/034).

### Role of the funding source

No funding source was utilized for this study.

## Results

### The profiles of pregnant women

Of the 2855 deaths reported to the MDSR from 2006 to 2018, 1646 (57.7%) were confirmed as maternal deaths (direct or indirect). Of those, 284 (17.25%) were attributed to HD complicating pregnancy.

The mean age of the women who died from HD was 30.0 (SD 5.9) years. There were 9 (3.2%) adolescents and 55 (19.4%) women older than 35.

Most of the women who died were Sinhalese, 184 (64.8%), and married 271 (95.4%). Most were educated up to the secondary level (208, 73.2%), and 46 (16.2%) were employed. Primigravida women accounted for 105 (37%) deaths.

More than 194 (68.3%) were registered at the antenatal clinic (ANC) on or before 12 weeks of gestation (POG). Only 51 (18%) failed to register at an ANC before 12 weeks. The place of management for 121 (58.4%) deaths was a tertiary care center.

Nearly one-third of women with HD (89, 31.3%), died during the antenatal period, and 3 (1.1%) died during the intra-natal period. However, most women (190, 66.9%) died after delivery. Of the women who died after delivery, 42 (22.1%) gave birth via an emergency lower segment cesarean section.

The primary pregnancy outcome of the women who died was a live birth of 156 (54.9%). A sizeable proportion (n = 106, 37.3%) died at a teaching hospital, while only 2 (0.7%) died at a primary care unit. A summary of the sociodemographic profiles of the women is provided in Table 1.

**Table 1:** The characteristics of pregnant women who died due to HD complicating pregnancy in Sri Lanka from 2006 to 2018.

	N (%)
**Age**	
<20	9 (3.2%)
21–25	62 (21.8%)
26–30	81 (28.5%)
31–35	76 (26.8%)
36–40	44 (15.5%)
>40	11 (3.9%)
**Ethnicity**	
Sinhala	184 (64.8%)
Tamil	55 (19.4%)
Muslim	41 (14.4%)
Other	1 (0.4%)
**Marital status**	
Married	271 (95.4%)
Unmarried	5 (1.8%)
Living together	2 (0.7%)
Separated	1 (0.4%)
Widowed	1 (0.4%)
**Education**	
Primary	33 (11.6%)
Secondary	208 (73.2%)
Tertiary	12 (4.2%)
None	7 (2.5%)
Unknown	24 (8.5%)
**Occupation**	
Occupied	46 (16.2%)
Non-occupied	233 (82.0%)
Unknown	5 (1.8%)
**Gravidity**	
Primigravida	105 (37%)
Multigravida	177 (62.32%)
**POA at registration to ANC**	
<12 weeks	194 (68.3%)
>12 weeks	51 (18.0%)
**Timing of death**	
AN	89 (31.3%)
IN	3 (1.1%)
PN	190 (66.9%)
**Mode of delivery in PN deaths (n = 190)**	
Vaginal delivery	25 (13.2%)
Assisted vaginal delivery	49 (25.8%)
EL-LSCS	33 (13.4%)
EM-LSCS	42 (22.1%)
Hysterotomy	4 (2.1%)
Abortion	8 (4.1%)
Missing data	27 (14.2%)
**Overall outcome of pregnancy**	
Live birth	156 (54.9%)
Still birth	18 (6.3%)
Abortion	24 (8.5%)
Not delivered	41 (14.4%)
Neonatal death	4 (1.4%)
Missing data	40 (14.1%)
**Place of overall management for hospital deaths (n = 207)**	
Tertiary care centre	121 (58.4%)
Secondary care centre	74 (35.8%)
Primary care centre	4 (1.9%)
Private hospitals	8 (3.9%)

**Fig. 2:**
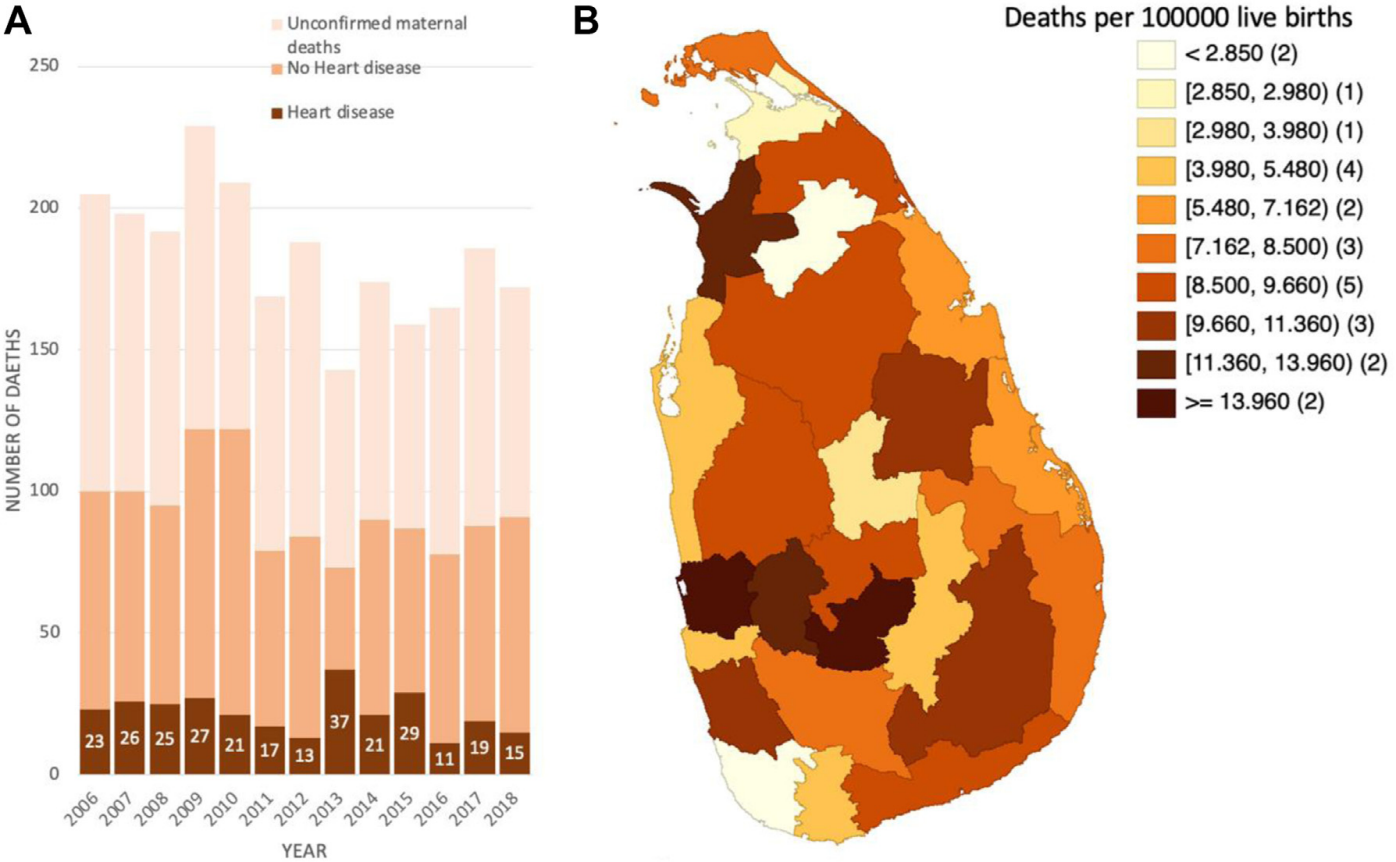
Temporal trends (A) and geographic distribution (B) of 466 HD-related deaths reported to the Sri Lankan maternal death surveillance system from 2006 to 2018.

The distribution of HD-related maternal deaths by year over the study period (Fig. 2A) varied from 11 (2016) to 37 (2013). The cause-specific maternal mortality ratio due to HD from 2006 to 2018 was 7.24 per 100,000 LBs. In addition, substantial subnational variations in HD-related deaths were observed (Fig. 2B), with less than 3 per 100,000 LBs in Galle, Kilinochchi, Matale, and Vavuniya, and 14.61 per 100,000 LBs in Nuwara Eliya district.

### Cause of death

**Fig. 3:**
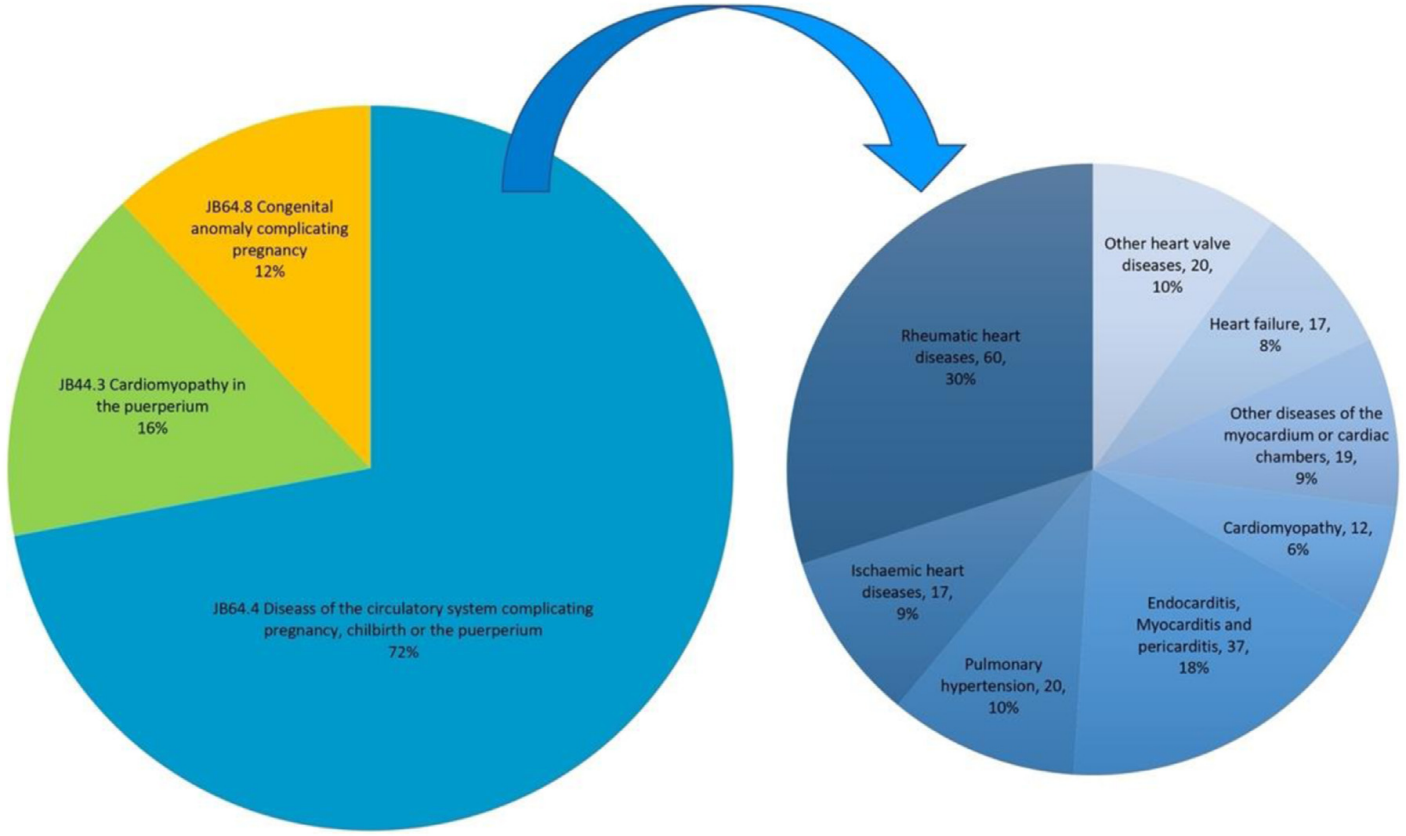
ICD-11 amalgamated classifications of HD-related deaths among pregnant women reported to the Sri Lankan maternal death surveillance system from 2006 to 2018.

Slightly more than two-thirds (204, 71.8%) of the deaths directly fell under the ICD 11–Pregnancy, childbirth, or the puerperium chapter, code JB64.4. Diseases of the circulatory system complicating pregnancy, childbirth, or the puerperium (Fig. 3) include rheumatic HD (60), other heart valve diseases (20), endocarditis, myocarditis or pericarditis (37), and pulmonary hypertension (20) as leading underlying causes. Cardiomyopathies in the puerperium (JB44.3, n = 46, 16.2%) and congenital anomalies (JB64.8, n = 34, 12%) were the other leading HDs identified.

Since the ICD 11 is for generic classification, the ICD 11–Pregnancy, childbirth, or the puerperium chapter codes were used for further classification. All entries were manually checked and separately for the entire dataset. Then, the categories were amalgamated for clinical interpretation and presented in Fig. 3. Cause-specific HD variation over the years is demonstrated in Supplementary Table S1.

Out of the 34 congenital anomalies reported, 10 (29.4%) were ventricular septal defects, and 12 (35.3%) were atrial septal defects.

The spatial distribution of rheumatic HD and congenital HD on the island is shown in Supplementary Figure S1. Congenital HDs are more prominent in the Mannar, Nuwara-Eliya, and Gampaha areas. On the other hand, rheumatic HD-related maternal deaths were more concentrated in Mannar, Monaragala, and Polonnaruwa.

### Health system determinants of maternal deaths due to cardiovascular conditions

Unmet need for family planning partially accounted for 97 (34.2%) deaths. Risk identification by the health system before death was made in 49.3% (n = 140) of cases, and 38 (13.4%) women had documented delays in risk identification. No risk factor was identified in 11 (3.9%) individuals before death. Factors related to the preventability of CVD-related maternal deaths in Sri Lanka are summarised in Table 2.

**Table 2:** Factors related to the preventability of CVD-related maternal deaths in Sri Lanka.

Variable	n (%)
**Unmet need of FP**	
Yes	97 (34.2%)
No	180 (63.4%)
**Risk condition identified**	
Yes, on time	140 (49.3%)
Yes, delayed	38 (13.4%)
Yes (unspecified)	27 (9.5%)
No	11 (3.9%)
Not relevant or missing data	64 (24%)
**Risk condition, follow-up**	
Done, adequate	123 (43.3%)
Done, inadequate	38 (13.4%)
Done, not specified	18 (6.3%)
No	1 (0.4%)
Not relevant or missing data	104 (36.7%)
**Medically contraindicated pregnancies**	
Yes	54 (19.0%)
No	218 (76.8%)
Missing data	12 (4.2%)
**Delay in care service**	
Yes	186 (65.5%)
No	35 (12.3%)
Inconclusive or missing data	62 (21.8%)
**Preventability decided by the review**	
Yes	158 (55.6%)
No	52 (18.3%)
Inconclusive or missing data	74 (26.0%)

Medically contraindicated pregnancies were identified as the possible cause of death among 54 (19.0%). All deaths were categorized in the reviews based on the 3-delay model.^19^ The 3-delay model is explained as the delay in the decision to seek care (first delay), the delay in arrival at a health facility (second delay), and the delay in the provision of adequate care (third delay).^19^ One or more delays leading to maternal deaths were identified in 186 (65.5%) individuals. The maternal mortality review process revealed that 158 (55.6%) of the deaths were preventable.

The place of death reported was the hospital (234, 82.4%), either upon admission or in transit (30, 10.6%), home (12, 4.2%), and on the way to the hospital 5 (1.8%). With adjustment to the LBs in the district, most of the home deaths occurred in the Gampaha district.

## Discussion

This data shows that heart diseases accounted for 17.3% of all maternal deaths in Sri Lanka, with a cause-specific annual maternal mortality rate of 7.24 per 100,000 live births. Rheumatic HD was the most common cause of death, and post-natal deaths accounted for the most fatalities. A significant percentage of these deaths were preventable, with delays in seeking, accessing, or providing care identified as contributing factors. These findings underscore the need for improved maternal healthcare services in Sri Lanka, particularly for women with hypertensive disorders during and after pregnancy.

The remaining high rheumatic HD in the country is an alarming indicator. Rheumatic HD is a leading killer under the HD category in places with poor overall care services, and in India, it has been shown to contribute to almost 70% of MMR due to HD.^20^ The Sri Lankan situation is far better, yet it underscores the urgent need to improve the country's overall socioeconomic and health status.

However, maternal mortality due to all valvular heart conditions, including rheumatic valvular HDs, accounted for the highest combined deaths (28.5%, n = 81). The high numbers of these conditions in Sri Lanka highlight an urgent need for all levels of care, including early detection in the field to identify valvular heart conditions.

With the obstetric transition and improved availability of primary care services, the underlying causes of maternal deaths in Sri Lanka and many other LMICs are transitioning, and indirect causes are increasingly high.^21^ The expansion of essential services and moving beyond routine service availability is required to further reduce maternal deaths. HD-related MMR in the Asian region varies from 3.2/100,000 in China to 19.7/100,000 in Bangladesh.^22^ However, in many upper-middle-income countries, HD-related MMR is less than 5/100,000, and in high-income countries, it is less than 2/100,000.^22^

Over 13 years, 31 (10.9%) undetected congenital malformations led to the deaths of pregnant or postpartum women, which could have been prevented if early screening, high-quality primary care services, and follow-up services were available. The contribution of congenital HD to maternal deaths varies across the globe. For example, a study from South Africa (2011–2013) showed that 5.1% of maternal deaths were due to congenital HDs (CHDs).^23^ Another study was conducted in 138 centers in 53 countries, with 3295 maternal patients with CHD; one-third of patients (n = 1059) had an uncorrected CHD.^24^ The life lifecycle approach in preventing maternal deaths should include screening for HD complicating pregnancy after birth, before discharge, in child welfare clinics, school health programs, and pre-conceptional clinics. In addition, the death of pregnant women in their second or subsequent pregnancies suggests that proper care during pregnancy and the post-partum period is crucial in preventing HD-related deaths in pregnancy.

Owing to global efforts, the prevalence of contraceptives increased from 54.8% to 63.3% from 1990 to 2010, reducing the unmet need for family planning from 15.4% to 12.3%.^25^ The estimated number of maternal deaths averted due to contraceptive use in 2008 was 272,040.^26^ Sri Lanka has a family planning prevalence of 67.7%, with 58.4% using modern methods.^27^ However, 34.2% of all HD-related maternal deaths were attributed to unmet family planning needs from 2006 to 2018. These included 54 medically contraindicated pregnancies. Whether these deaths were due to issues of availability, acceptability, affordability, or knowledge, the impact on family planning services needs to be explored appropriately, and these numbers clearly show a need for further improvement of family planning services in Sri Lanka.

One primary concern that emerged from these data is gross subnational discrepancies in HD-related deaths. These numbers demonstrate inequity in health, primarily associated with education, social status, and access to and availability of healthcare services. High maternal mortality rates and poor general health indicators in the Nuwara Eliya district have long been known to Sri Lankan health officials.^28^ Despite interventions to improve access to health care by infiltrating estates through the medical officer of the health system, a lack of education, poverty, and unhealthy sociocultural behaviors (including delayed care-seeking practices) lead to poor outcomes in the estate sector. In addition, transport and service availability are challenges. A similar setup still prevails in the Mannar district.^15^ Reorientation of the health care system is needed to identify pockets with high MMR and focused programs to improve care provision. The subnational disparities observed in Sri Lanka are common across LMICs and HICs. A previous study in India showed that 85.5% of maternal deaths occurred in rural settings.^29^ Similarly, a study conducted in the US from 2013 to 2017 showed 18.8 and 33.8 maternal deaths per 100,000 LBs in large central metro regions and small rural areas, respectively.^30^

These data underline the need for strategic changes in maternal care in Sri Lanka to reduce maternal deaths further. The prevention of HD as a leading cause of maternal deaths requires the lifecycle approach with better care of newborns, school children, and adolescent girls, pre-conceptional care, situation-specific interventions, and highly specialized care. Specific strategies need to be developed using the available evidence. Community awareness, capacity building regarding management, and specific infrastructure development will be key strategies. However, context-specific factors determining HD mortality within the country require focused research to identify non-medical root causes linked to HD mortality among pregnant and post-partum women, specifically in areas with high HD-related MMR.

ICD classification was performed using available data on maternal death investigations. Missing data were unavoidable from routine field investigations.

## Contributors

Conceptualization: AH, SA, KJ.

Methodology: KJ, AH, SA.

Data curation: AH, SA, KJ.

Formal analysis: AH, SA, KJ.

Writing- original draft: AH.

Writing-review and editing: AH, KJ, CDS, HS, NL, SA.

Supervision: CDS, HS, NL, SA.

## Data sharing statement

No new primary data were collected for this study. Data is owned by the Family Health Bureau, Ministry of Health Sri Lanka. Requests for further use can be directed to the National Programme Manager–Maternal & Child Morbidity & Mortality, Dr. Kapila Jayarathne (kapjay613@gmail.com), at the Family Health Bureau, Ministry of Health Sri Lanka.

## Editor note

The Lancet Group takes a neutral position with respect to territorial claims in published maps and institutional affiliations.

## Declaration of interests

None declared.
