# Supplementary material for: Heart disease complicating pregnancy as a leading cause of maternal deaths in LMIC settings: the Sri Lankan experience
Source: Lancet Reg Health Southeast Asia. 2023 Jun 6;15:100223. doi: 10.1016/j.lansea.2023.100223 (PMC10442957; doi:10.1016/j.lansea.2023.100223)
Supplement: Supplementary Figure and Table [file mmc1.pdf]

Content	Page number
Supplementary Figure 1	2
Supplementary Table 1	3

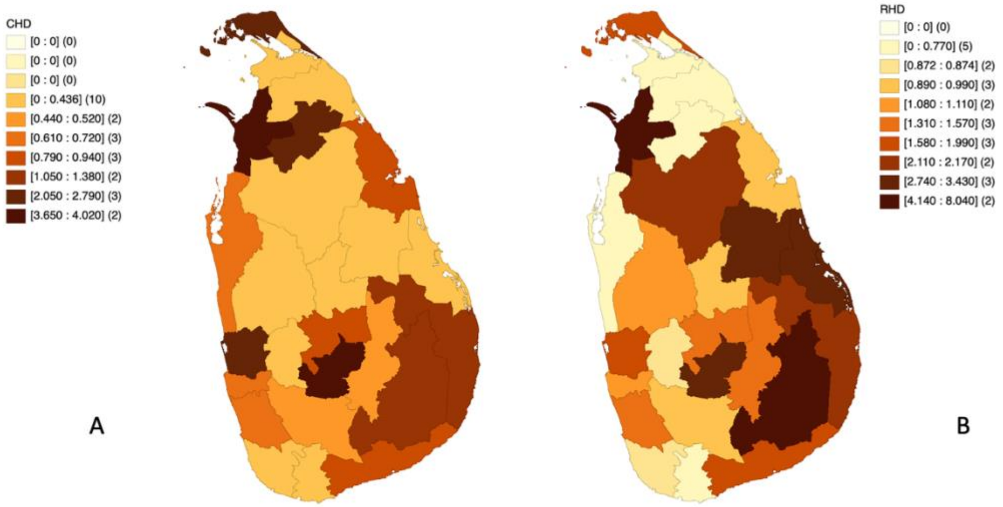

Supplementary Figure 1 - Geographic distribution of congenital HD (A) and rheumatic HD (B)-related maternal deaths reported to the Sri Lankan maternal death surveillance system from 2006 to 2018.

Supplementary Table 1. The variation of cause-specific maternal deaths due to individual conditions classified under heart disease complicating pregnancy (2006-2018)

	**2006-2009**	**2010-2013**	**2014-2018**
Rheumatic HD	12 (11.9%)	27 (31%)	21 (21.9%)
Ischaemic HD	7 (6.9%)	3 (3.4%)	7 (7.3%)
Pulmonary hypertension	5 (5%)	6 (6.9%)	9 (9.4%)
Endocarditis, myocarditis, & pericarditis	19 (18.8%)	11 (12.6%)	7 (7.3%)
Cardiomyopathy	15 (14.9%)	13 (14.9%)	11 (11.5%)
Other diseases of the myocardium or cardiac chambers	5 (5%)	6 (6.9%)	8 (8.3%)
Heart failure	16 (15.8%)	0	2 (2.1%)
Heart valve diseases	9 (8.9%)	4 (3.4%)	9 (9.4%)
Structural developmental anomalies of the circulatory system (congenital)	9 (8.9%)	11 (12.6%)	11 (11.5%)
Cardiomyopathy in the puerperium	3 (3%)	7 (8%)	10 (10.4%)
Myxoma	1 (1%)	0	1 (1%)
